# Aftermath

**Published:** 1896-09

**Authors:** 


### AFTERMATH.

It was good to have been there.

Chairman Hinkley proved a tireless worker, and everyone was more than delighted

Messrs. McLeod, Somerville and Gangloff seemed to be always where they were needed, and their zeal in looking after the comfort and pleasure of the delegates and their friends was ever uppermost in their minds.

The re-election of that faithful watch-dog of the treasury to the twelfth term was a strong evidence of the honor and respect that the Association has for its faithful member, James L. Robertson.

Handsome badges were provided for all, and especially beautiful were those selected for the President and Secretary.

Not even the need of a gavel to call the members to order was forgotten, and the outgoing President very thoughtfully presented it to his successor as a token of the kindness of the Buffalo veterinarians, with the sincere wish that its gentle raps would win for him the same kindly attention, consideration, and support that it had brought to the retiring officer in presiding over the deliberations of the thirty-third annual gathering.

Acting Mayor Boeckel, in conveying to the delegates the freedom of the city, made many thoughtful suggestions appreciative of the importance of our work and its close alignment with successful city government in providing for the people in crowded centres that second great necessity—a pure milk-supply.

Plenty of good water at the Convention hall, a bureau of information at the hotel headquarters, and members of the local committee of arrangements on every side were some of the thoughtful provisions made for our comfort and entertainment.

Journalistic enterprises were well represented by Editors Bell, Pearson, Gill, Berns, and Hoskins.

At one time representatives of eleven colleges could be noted on the seats of the Convention hall.

The decorations at the Genesee Hotel and the placards of the Convention programme at the hotel and university were thoughtful suggestions, and the latter were fruitful of much saving of the officers' time and a great convenience to the delegates.

Our second day was ushered in with strains of beautiful music that hovered around us on our trolley and boat ride and back to our chosen city. This feature of our entertainment was furnished by one of Buffalo's finest bands.

Agricultural Experiment Stations were more than well represented, and they took the initial steps toward an affiliated organization of their representatives.

Section work in our national organization is rapidly making itself felt as a future necessity, and no doubt will greatly enhance the power of the Association throughout the land.

Alabama was represented, alike Minnesota, Nebraska, New Hampshire, Vermont, and Connecticut, with a strong contingent from Massachusetts.

Iowa, Kansas, Tennessee, Michigan, Maryland, Ohio, and Illinois sent representatives of their States to speed the good work.

The provinces of Quebec and Ontario were there, too, and soon the question of changing our name to American Veterinary Medical Association will be a leading question.

"He was a good square fellow," so said the conductor of a Buffalo trolley car when he was handed a nickel by one of the delegates, who. in his haste to stop the car as he was being hurried by his hotel, rang up a fare instead.

The Gorge trolley-ride and the steamer-trip down the lake, with the bounteous spread of all the good things and the kindness of our friends, will never be forgotten.

The largest and most representative convention ever held in the United States.

Manitoba was well represented. Montana had her quota; Lousiana one of her strong devotees of the profession, and two more of her veterinarians have entered the ranks.

The probationary period of six months for all Bureau of Animal Industry inspectors seems a wise provision, and enables heads of departments or stations to judge of the relative qualifications of new appointees and to relieve those from duty who are unfitted for the service, without any stigma thus resting against them.

The disposition of all the members of the U. S. V. M. A. to obey its mandates augurs well for the profession, and is an acknowledgment of the high standing of the Association in veterinary circles.

We are glad to note that so many of the members and the profession so fully appreciate the fact that, whether they are able to attend the Convention meetings or not, it is a duty to contribute to its work and power to do good by joining and adding their initiation fees and dues to the fund which is used in behalf of the whole profession.

Member Wende, of the Committee on Diseases, was much relieved when he had waded through such a mass of important though ofttimes amusing statistics of the prevalence of disease.

President Osgood well merits the honor accorded him, and fully appreciates the responsibilities of such a charge. His broad ideas, his high conceptions of the value of association work well equip him to lead us on to higher work, better attainments, and a stronger organization.

The recognition of Drs. R. R. Bell, M. R. Trumbower, and M. Stalker for Eastern, Central, and Western Vice-Presidents respectively places a strong trio to aid the chief executive.

The Army Legislative Committee will have added Dr. Austin Peters, of Boston, Mass., to aid Messrs. J. P. Turner and John R. Hart. This important work rests safely in their hands.

Prof. A. Smith, of Toronto, proved a royal entertainer of those who had the pleasure of the trip to Canada and the Industrial Exposition in that city.

Under a suspension of the rules suitable recognition was made of the death of Dr. John W. Gadsden, and resolutions of respect were placed upon the records of the Association.

The Publication Committee had its own way all through, and the newly elected President again placed this important work in the hands of Chairman Williams and his old assistants, Messrs. Hinkley and Stewart.

Too much praise cannot be given Secretary Stewart for his successful work in behalf of the Association during the past year.

The prospects of a better filled treasury will greatly aid the new officers and strengthen the Association's hands for renewed effort.

Potted plants, choice vessels filled with beautiful flowers, and gracefully arranged sweet-peas woven in a green background around the entire banquet-table were beautiful to the eye, and an elegantly prepared *menu* of tempting and appetizing dishes greeted the keen and appreciative appetites of fifty-eight of our number as a fitting close to our deliberations at the Genesee Hotel.

Hands across the sea were very suggestive in the missed snap-shot camera scene at the head of the banquet-table when those warriors of veterinary education, Messrs. Smith and McEachran, grasped hands over the future of higher veterinary work in North America. It was good to see them there; but there was one other missed to complete the scene, viz., our much-loved member, Prof. Liautard.

We will play that game of cards some other day when the lake is not so rough and the fishes not so hungry.

I won't lie down there, for it looks too much like a morgue. Cut my head off.

Only three good sailors in the party; but then it was very rough, and chocolates were not suitable food. What a dismal sound came from the supper-bell.

Ever going southward, it was not surprising that the way to the hotel early in the morning seemed in the wrong direction.

The Buffalo University building proved a very suitable place for the Convention.

Those who elect to conduct a boom in the interest of their friend should go to bed earlier and not reach the arena of battle an hour and a half after the appointed time for the election.

I would like to be your Trilby, even though you are a Svengali and a Czar Reed.

The lady visitors, larger in number than ever before, will never forget their trip to the "Queen City of the Lakes" and the unceasing kindness and courtesies tendered them by Mrs. Nelson P. Hinkley.

The Keystone State was well represented, and high was their praise of the kindness of their colleagues in and around Buffalo.

Member Hart, of the Army Legislative Committee, made a strong appeal for help for his committee, and vouchsafed the promise that success would crown their efforts at the next session of Congress.

Messrs. James Robertson, McKenzie, and McAnulty made strong pleas for help and recognition for the work among the horseshoeing craft, and, though a wide diversity of opinion prevailed, resolutions of encouragement were subsequently adopted with but two dissenting voices.

The Buffalo *Horse World* of September 4th contained a very good account of our convention.

Between Nashville, Tenn.; Columbus, O.; Detroit, Mich.; Kansas City, Mo.; and Cleveland, O., there is sure to be considerable rivalry for the convention of 1897. It is a wholesome sign of prosperity.

Buffalo's reception and entertainment of the thirty-third annual convention surpassed all affairs of a similar nature, and earned for them the reputation of royal entertainers.

Much has been said, a great deal can be said, but the least said was the final conclusion in the Association of Faculties and U. S. V. M. A. Committee as to past differences of opinion as to the proper organization of the former body, and this conclusion makes easier the mending of any breaks in the Association fabric. Its report was accepted and all acquiesced in its acceptance, and the utmost harmony prevailed in the deliberations of this body, at which some eleven veterinary schools were represented. Many features of future teaching and requirements for matriculation were discussed, and from it is hoped a single plan may be devised for carrying out a single matriculation examination.

